# Clinical aspects of cardiolaminopathies and prospects for a cardiolaminopathy registry

**DOI:** 10.1186/1750-1172-10-S2-O30

**Published:** 2015-11-11

**Authors:** Sara Benedetti

**Affiliations:** 1 Laboratory of Clinical Molecular Biology and Cytogenetics, San Raffaele Scientific Institute, Milano, Italy

## 

Mutations in the *LMNA* gene, encoding nuclear proteins lamin A/C, have been associated with neurological and cardiac disease and a high risk of sudden death. The implant of a cardioverter defibrillator (ICD) is to date the only effective intervention, but no specific guidelines are available. We decided to create a common Italian database integrating clinical and genetic data of patients bearing *LMNA* gene mutations to improve knowledge of natural history of cardiopathy, define a risk stratification protocol for ICD implant and investigate genotype/phenotype correlations.

To date, 113 patients (age 47±18) from 11 Italian centers have been included in our database and followed for 7±11 years. We evaluated age at onset of different phenotypes. 70% developed cardiac symptoms, including both rhythm (atrial fibrillation, atrio-ventricular block, ventricular tachycardias) and structural defects (dilated cardiomyopathy, mitral insufficiency), which may or may not be preceded by neurological signs. Cardiac magnetic resonance was pathologic in 2/3 of studied patients. We also evaluated the occurrence of ICD implantation, appropriate shocks, cardiac transplantation and heart failure. Open questions include the identification of predictors of arrhythmias to allow early diagnosis and improve risk stratification and management of asymptomatic patients.

